# Long-term outcomes of the international EXPAND trial of Organ Care System (OCS) Lung preservation for lung transplantation

**DOI:** 10.1016/j.eclinm.2025.103334

**Published:** 2025-07-08

**Authors:** Gabriel Loor, Gregor Warnecke, Mauricio A. Villavicencio, Michael A. Smith, Xiang Zhou, Jasleen Kukreja, Abbas Ardehali, Matthew G. Hartwig, Mani Ali Daneshmand, Marshall I. Hertz, Stephen J. Huddleston, Axel Haverich, Joren C. Madsen, Arne Neyrinck, Dirk Van Raemdonck

**Affiliations:** a Division of Cardiothoracic Surgery, Michael E. DeBakey Department of Surgery, Baylor College of Medicine, Houston, TX, USA; b Department of Cardiovascular Surgery, The Texas Heart Institute, Houston, TX, USA; c Department of Cardiac Surgery, University Schleswig-Holstein (UKSH) Campus Kiel, Kiel, Germany; d Department of Cardiovascular Surgery, Mayo Clinic, Rochester, MN, USA; e Department of General Thoracic Surgery, St. Joseph's Medical Center, Phoenix, AZ, USA; f TransMedics Inc, Andover, MA, USA; g Division of Cardiothoracic Surgery, University of California San Francisco, San Francisco, CA, USA; h Division of Cardiothoracic Surgery, Department of Surgery, Ronald Reagan University of California, Los Angeles Medical Center, Los Angeles, CA, USA; i Division of Cardiovascular and Thoracic Surgery, Duke University Medical Center, Durham, NC, USA; j Division of Cardiothoracic Surgery, Emory University School of Medicine, Atlanta, GA, USA; k Department of Pulmonary, Allergy, Critical Care and Sleep Medicine, University of Minnesota, Minneapolis, MN, USA; l Division of Cardiothoracic Surgery, University of Minnesota, Minneapolis, MN, USA; m Department of Cardiac, Thoracic, Transplantation, and Vascular Surgery, Hannover Medical School, Hannover, Germany; n Massachusetts General Transplant Center and Department of Cardiac Surgery, Massachusetts General Hospital, Boston, MA, USA; o Department of Anaesthesiology, University Hospitals Leuven, Belgium; p Department of Thoracic Surgery, University Hospitals Leuven, Leuven, Belgium

**Keywords:** Ex vivo lung perfusion, Lung transplantation, Bronchiolitis obliterans syndrome, Chronic lung allograft dysfunction, Primary graft dysfunction, Donation after circulatory death

## Abstract

**Background:**

Portable ex vivo lung perfusion and ventilation with the Organ Care System (OCS) Lung system is a safe, effective method for preserving extended criteria donor (ECD) organs before transplant. Although this technology is increasingly used in the United States, no published data describe its effects on long-term graft function and patient outcomes. This study assessed long-term clinical outcomes after transplantation of ECD lungs that were preserved, recruited, and assessed with the OCS Lung.

**Methods:**

The EXPAND Lung Trial was a prospective, single-arm, multicenter, international trial conducted between January 2014 and July 2016; 5-year follow-up data were collected until December 2021. Double-lung donors were included who met any of four ECD criteria: age ≥55 years, PaO_2_/FiO_2_ ≤300 mmHg, expected ischemic time >6 h, and donation after circulatory death (DCD). Transplant recipients’ overall survival and 5-year incidence of bronchiolitis obliterans syndrome (BOS) were compared between the EXPAND cohort (n = 79) and a control cohort from the same centers within the same time period, who received donor lungs preserved with ice but not OCS (n = 644). This study is registered with ClinicalTrials.gov (NCT04194398).

**Findings:**

Overall survival was similar between the EXPAND and control cohorts; 5-year overall survival was 68.1% versus 66.5%, respectively (*P* = 0.795). The risk factors associated with overall survival were the degree of urgency for lung transplant and recipient age; 5-year survival was much greater for patients designated as non-urgent than for patients designated as urgent (73% versus 41%, *P* = 0.021). 5-Year BOS3-free survival was 60.4% for the EXPAND cohort and 63.7% for the control cohort (*P* = 0.599). Overall survival, development of BOS3, and development of any grade of BOS did not differ between the EXPAND and control cohorts.

**Interpretation:**

Among patients who underwent lung transplantation with ECD lungs, the use of OCS Lung resulted in excellent long-term clinical outcomes. This study's findings support the use of OCS Lung to expand the donor pool and provide a foundation for future studies comparing lung-preservation strategies.

**Funding:**

This study was funded by 10.13039/100031958TransMedics.

**Research in context**

**Evidence before this study**

Donor organ preservation is currently a major area of innovation in transplantation, and for good reason—it can increase the number of viable donor organs to better meet the demand. In lung transplantation, portable ex vivo lung perfusion (EVLP) using the Organ Care System (OCS) Lung has been the most rigorously studied organ preservation strategy in lung transplantation. A large randomized controlled clinical trial (INSPIRE) associated the use of the OCS with reduced primary graft dysfunction, and a pivotal single-arm clinical trial (EXPAND) showed excellent early results with extended criteria donor organs. This led to the US Food and Drug Administration (FDA) approval of the OCS for donor organ preservation. Despite these promising early results, no studies have yet been published regarding the *long-term outcomes* associated with OCS Lung preservation. This information is of critical importance to the lung transplant community due to the high incidence of chronic rejection. In fact, no long-term outcomes of FDA-approved organ-preservation devices are reported in the literature. The only published long-term EVLP data come from a single-center study by Divithotawela et al., who used the Toronto General protocol for static EVLP. The results of this study were promising, but no multicenter evaluation of this approach has been published.

**Added value of this study**

The present study, an extension of the EXPAND pivotal trial, examined 5-year follow-up data from a unique, multicenter, prospective cohort of patients who received an extended donor lung preserved with OCS Lung. These lungs had been turned down for transplant by numerous centers. The results of this study suggest that these patients’ long-term outcomes were excellent and equivalent to those of a benchmark cohort of transplant recipients who received lungs preserved by standard methods. This finding is of critical importance to lung transplant surgeons, because it provides confidence that lungs preserved with the OCS Lung can produce outcomes as good as those of ice-preserved lungs.

**Implications of all the available evidence**

The results of this study provide high-level evidence that OCS Lung, and EVLP in general, can be used to preserve extended criteria donor lungs without a penalty in long-term outcomes for recipients. The findings also suggest the possibility that OCS Lung preservation makes extended criteria lungs as safe for patients as ice-preserved standard-criteria lungs, although this will require further investigation.

## Introduction

Candidates for lung transplantation face significant donor shortages and suboptimal waitlist survival.^1^ These limitations could be addressed by using lungs procured from extended criteria donors (ECDs), including donors with older age, low oxygenation, prolonged ischemic times, and donation after circulatory death (DCD).^2^, ^3^, ^4^, ^5^, ^6^ However, ECD lungs are underutilized because of concerns about ischemia-induced acute lung injury and lack of functional assessment and rehabilitation tools for the allograft.^1^^,^^3^^,^^5^^,^^7^

To overcome these concerns, ex vivo lung perfusion (EVLP) was conceived to enable the assessment and reconditioning of ECD lungs before transplantation.^4^^,^^6^^,^^8^ The Organ Care System (OCS) Lung™ is a portable EVLP platform that enables normothermic blood-based perfusion and ventilation of donor lungs throughout storage and transportation. Unlike a static (ie, non-transportable) EVLP system, this system could potentially begin reconditioning the lung early, at the donor hospital, while eliminating cold ischemia throughout the transportation process. In a previous trial (Prospective, International, Multi-Center, Randomized Clinical Investigation of TransMedics® Organ Care System for Lung Preservation and Transplantation; INSPIRE), using OCS Lung to preserve standard-criteria donor (SCD) lungs was associated with a lower incidence of early primary graft dysfunction (PGD) than using standard cold storage.^9^ Subsequently, in the International Trial to Evaluate the Safety and Effectiveness of the Portable Organ Care System Lung For Recruiting, Preserving and Assessing Expanded Criteria Donor Lungs for Transplantation (EXPAND), using OCS Lung to preserve ECD lungs was associated with 87% utilization of the ECD allografts and excellent early outcomes (91% 1-year survival).^10^ The rate of severe PGD (PGD3) at 72 h was 6%, which was lower than the 28–29% rates found in other studies of static EVLP for ECD lungs.^11^^,^^12^ However, the rate of PGD3 at any time point within 72 h, including baseline assessments made 6–8 h after reperfusion, was 44% in the EXPAND OCS cohort. This was higher than the 18% observed in the INSPIRE OCS study, which used SCD lungs, but lower than the 88.9% observed in the Develop UK static EVLP study, which used ECD lungs.^9^^,^^11^

The purpose of this study was to examine long-term outcomes of patients who received OCS-preserved allografts in the EXPAND trial by analyzing 5-year follow-up data. These 5-year outcomes were benchmarked against those of contemporary patients who received lungs preserved with standard ice preservation at EXPAND trial study sites.

## Methods

### Study design

This prospective, observational follow-up study of the original EXPAND cohort is registered with ClinicalTrials.gov (NCT04194398). Site Institutional Review Board and Ethics Committee approvals were obtained, and patients were consented per protocols for continued review of the EXPAND cohort. To increase the rigor of this study and benchmark these long-term outcomes, a secondary analysis was performed with a control group of transplant recipients treated at six of the eight participating institutions over the same time period. These control data were supplied by the United Network for Organ Sharing (UNOS) as the contractor for the Organ Procurement and Transplantation Network (OPTN). The interpretation and reporting of these data are the authors’ responsibility and in no way should be seen as an official policy of or interpretation by the OPTN or the US government. A historical comparison group is commonly used in reports of EVLP with ECD organs because of the ethical questions associated with randomizing ECD organs to ice versus EVLP.^11^^,^^13^^,^^14^

### Cohorts

The EXPAND trial was a single-arm study conducted at eight academic transplant centers—six in the United States, one in Germany, and one in Belgium—between January 2014 and July 2016; 5-year follow-up data were collected until December 2021. Details of the EXPAND study design were previously published.^10^ Double-lung donors were included if they had any of the following risk factors: age ≥55 years, PaO_2_/FiO_2_ (PF) ≤300 mmHg, expected ischemic time >6 h, and DCD donor. Less than 35% of lung transplants performed worldwide use donor lungs with these criteria; thus, such lungs were referred to as ECD lungs in the EXPAND trial.^2^^,^^3^^,^^13^^,^^15^, ^16^, ^17^ Lungs were perfused and ventilated on OCS from procurement until transplantation and were physiologically monitored continually as described previously.^9^ Lungs were deemed eligible for transplantation if three criteria were met after OCS preservation: 1) vascular resistance, pulmonary artery pressures, or peak airway pressures within 20% of starting values; 2) PF > 300 mmHg; and 3) confirmation by the transplanting surgeon of clinical suitability for transplant.

**Fig. 1:**
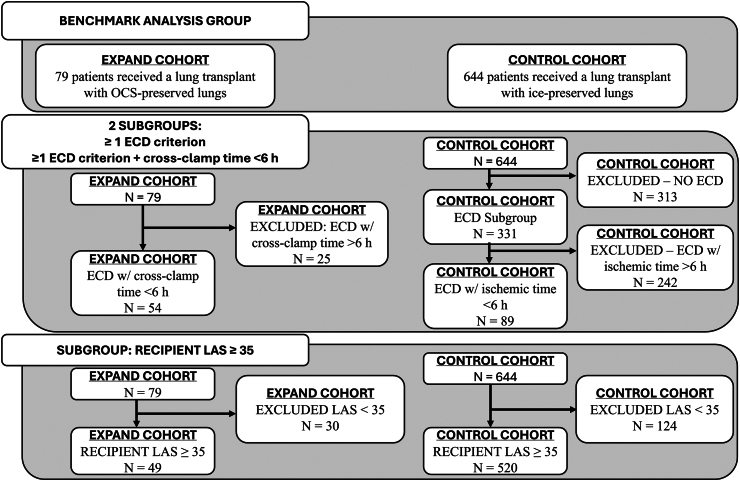
Flowchart of patient recruitment and assignment to groups. ECD extended criteria donor, LAS lung allocation score, OCS Organ Care System.

The control group comprised consecutive recipients of primary, double-lung transplant (recipient age ≥18 years) during the same period (Jan 2014–Jul 2016) at the six US institutions, where the allografts were preserved by standard ice (ie, non-OCS) methods. Similar control data were not available from the two European centers. As in the EXPAND cohort, donors with multiple transfusions (>10 packed red blood cell units), ABO incompatibility, or a >20 pack-year smoking history, as well as recipients with a prior solid-organ transplant, multi-organ transplant, or pretransplant use of dialysis, were excluded from the control cohort. Certain EXPAND exclusion criteria (moderate to severe lung injury, confirmed active pneumonia, persistent purulent secretions, history of pulmonary disease, and recipient diagnosis of chronic renal insufficiency) were not available in the UNOS data. Donors among the non-OCS controls who met one or more of the ECD criteria were used as a subgroup of controls. For the control patients, only actual ischemic time was available, which differs from expected ischemic time; hence, this subgroup of controls was examined both with and without the risk factor of expected ischemic time >6 h (Fig. 1).

### Outcomes

The primary endpoints were overall survival and freedom from bronchiolitis obliterans syndrome (BOS). Each center determined the presence or absence of BOS in their patients. BOS was suspected if the patient had a delayed and sustained drop in forced expiratory volume in 1 s (FEV_1_) below baseline values.^14^ The BOS diagnosis was ultimately based on the transplant physicians’ best judgment after considering non-immunologic reasons for the decline. BOS grade 3 (BOS3) was defined as a sustained decline in FEV_1_ to ≤50% of baseline.

The 2019 update of the International Society for Heart and Lung Transplantation (ISHLT) guidelines for reporting long-term graft dysfunction suggests using a slightly different definition, termed “chronic lung allograft dysfunction” (CLAD), rather than BOS.^18^ However, BOS was the prespecified endpoint at the start of the EXPAND trial in 2014 and thus was used for the current analysis. Data on patient immunosuppression, acute rejection, and treatment of rejection were not collected by the study sites and therefore were not included in this study.

### Statistical analysis

Time-varying overall survival and freedom from BOS were compared between groups by the Kaplan–Meier method, with statistical significance defined as log-rank *P* < 0.05. For the analysis of overall survival, the event was death, and patients were censored at the last visit date if death had not occurred. For the freedom-from-BOS analysis, the event was any BOS 1, 2, or 3 (whichever occurred earliest), and patients were censored at the last visit date (or death) if they did not have BOS. For the freedom-from-BOS3 survival analysis, the event was BOS3 or death (whichever occurred earlier), and patients were censored at the last visit date if alive without BOS3. In addition to the Kaplan–Meier comparisons, we used the Fine and Grey method to test BOS3 as the event of interest between the OCS and control cohorts by setting death as the competing risk factor. *P*-value was derived from a hazard ratio Cox regression model.

To identify clinical factors associated with overall survival, donor and recipient factors were assessed, including postoperative PGD scores within 72 h and urgency of transplant. For the current analysis, we treated urgency of transplant as a binary covariate. An urgent recipient was defined as one with a lung allocation score (LAS) ≥50 for the US and German sites and a designation of “high urgency” for the Belgian site. This was necessary because the US and German centers documented and collected individual LAS scores for the study according to the 2015 model for calculating LAS,^19^ whereas the Belgian center did not. Generally, US recipients with an LAS score ≥50 and European recipients with high urgency status have a greater risk of death without a transplant, are more likely to be hospitalized, and are more likely to be critically ill than recipients with LAS <50 or non–high-urgency status.^1^^,^^19^ We used the Kaplan–Meier method (log-rank test) and Cox regression analysis (multivariable analysis) to test for associations between clinical factors and overall survival. In our univariate analysis, we tested any potential risk factors for 5-year mortality. We then selected statistically significant risk factors with *P* value < 0.05 or risk factors that were clinically relevant with sufficient sample size and we entered these variables into the final Cox model to calculate the hazard ratio. We also performed propensity-score matching to compare 5-year overall survival between similar patients in the EXPAND and control cohorts.

All outcomes were summarized with descriptive statistics, specifically the mean (standard deviation) and range for the continuous variables and the proportion for the categorical variables. *P*-values were calculated using the two-sample t-test for continuous variables and the Fisher Exact test for categorical variables unless otherwise specified. All analyses were done with SAS, version 9.4.

### Role of the funding source

The funder of the study (Transmedics) designed the study in collaboration with the FDA and collected the data. While the study investigators had access to the deidentified dataset, a Transmedics employee (Xiang Zhou, PhD) performed the analyses for this study. Transmedics had no role in writing the report.

## Results

### Recipient and donor characteristics

The EXPAND trial has been previously described.^10^ Briefly, 93 donor lung pairs were retrieved. Twelve of these were excluded after OCS perfusion for a variety of clinical reasons, including persistent purulent secretions, edema, unstable OCS parameters, and lung damage. Eighty-one donor lung pairs met transplant criteria, resulting in a donor lung utilization rate of 87%. Two of these lung pairs were not transplanted for logistical reasons. Thus, the final EXPAND cohort comprised 79 patients who received transplanted lungs that had been preserved by and assessed on the OCS Lung System.^10^ The benchmark control cohort comprised 644 patients who received transplanted lungs preserved with standard static ice preservation methods. As expected, OCS donors were older (47 versus 37 years), and their allografts had longer total cross-clamp times (609 versus 342 min) but shorter total cold ischemic times (235 versus 342 min) than the controls. In general, EXPAND patients were more likely than control patients to have ECD characteristics (donor age ≥55 years, PF ratio ≤300, anticipated cross-clamp time >6 h, and DCD) (Table 1). In addition, the mean PF ratio was 378 for the OCS group and 436 for the control group (*P* < 0.0001). The incidence of abnormal findings on bronchoscopy was 65% in the OCS group and 30% in the control group (*P* < 0.0001).

**Table 1:** Donor and recipient characteristics compared between the EXPAND Organ Care System and standard-of-care control cohorts.

	Statistics	EXPAND (OCS) N = 79	Controls (SOC) N = 644	*P*
Donor demographics				
Age, years	mean ± std (min–max)	47.1 ± 16.2 (16.7–76)	36.9 ± 15.0 (9–77)	<0.0001
Sex–female	n/N (%)	33/79 (41.8%)	243/644 (37.7%)	0.5398
BMI, kg/m^2^	mean ± std (min–max)	27.1 ± 5.9 (16.9–44.1)	26.7 ± 6.3 (16.1–66.0)	0.6172
Total CC time, mins	mean ± std (min–max)	609.4 ± 127.5 (353–1047)	341.8 ± 100.6 (110–900)	<0.0001
Total ischemic time, mins	mean ± std (min–max)	235.0 ± 95.2 (57–436)	341.8 ± 100.6 (110–900)	<0.0001
Donor risk factors				
Expected CC time >6 h	n/N (%)	25/79 (31.7%)	242/643 (37.6%)^a^	0.3250
DCD donor	n/N (%)	26/79 (32.9%)	21/644 (3.3%)	<0.0001
PaO_2_/FiO_2_ ratio ≤300 mmHg	n/N (%)	20/79 (25.3%)	56/642 (8.7%)	<0.0001
Donor age ≥55 years	n/N (%)	31/79 (39.2%)	96/644 (14.9%)	<0.0001
>1 risk factor	n/N (%)	21/79 (26.6%)	78/644 (12.1%)	0.0014
PaO_2_/FiO_2_ ratio	mean ± std (min–max)	377.7 ± 110.0 (135.0–663.0)	435.6 ± 119.3 (56.0–1497)	<0.0001
Abnormal finding from imaging	n/N (%)	51/79 (64.6%)	431/644 (66.9%)	0.7050
Abnormal finding from bronchoscopy^b^	n/N (%)	51/79 (64.6%)	193/644 (30.0%)	<0.0001
Recipient demographics				
Age, years	mean ± std (min–max)	55.6 ± 10.6 (31.8–73.7)	54.7 ± 13.3 (20.0–76.0)	0.5024
Sex–female	n/N (%)	33/79 (41.8%)	261/644 (40.5%)	0.9035
BMI, kg/m^2^	mean ± std (min–max)	24.5 ± 4.6 (16.2–33.6)	24.8 ± 4.3 (15.5–36.1)	0.5092
LAS at transplant	mean ± std (min–max)	42.0 ± 13.5 (31–93)	50.0 ± 18.7 (28.4–95)	<0.0001
Recipient medical history				
Diabetes	n/N (%)	19/79 (24.1%)	127/644 (19.7%)	0.3739
Pretransplant ECMO	n/N (%)	1/79 (1.3%)	50/644 (7.8%)	0.0332

BMI body mass index, CC cross-clamp, DCD donation after cardiac death, ECMO extracorporeal membrane oxygenation, LAS lung allocation score, OCS Organ Care System, SOC standard of care.

a For the control cohort, the cross-clamp time represents the actual rather than the expected cross-clamp time.

b Abnormal findings from bronchoscopy included purulent secretions, unusual anatomical structures or lesions not commonly observed in donors, and signs of aspiration. These signs of aspiration included significant erythema indicative of chemical irritation, as well as visible bile, blood, or other aspirated materials. *P*-values were calculated with the two-sample t-test for continuous variables and the Fisher Exact test for categorical variables.

Regarding DCD donors, OCS and control patients did not differ in donor age, donor BMI, PF ratio, or warm ischemia time (Supplementary Table S1). The average warm ischemia time (from withdrawal of life support to pulmonary artery flush) was 27.6 ± 4.8 min for the OCS group and 27.8 ± 8.4 min for the control group (*P* = 0.92). While there was no limit for warm ischemic time in the EXPAND trial, given the novelty of the device, most centers were not willing to exceed 60–90 min. Total cross-clamp time was 607.9 ± 146 min for the OCS group and 346.4 ± 96.7 min in the control group (*P* < 0.0001).

Notably, recipients in the EXPAND cohort had significantly lower mean LAS (42.0 ± 13.5 versus 50.0 ± 18.7; *P* < 0.0001) and a lower incidence of pretransplant extracorporeal membrane oxygenation use (1.3% versus 7.8%; *P* = 0.0332) than the control cohort. These differences were further explored in several subgroup analyses, as detailed below.

### EXPAND cohort: survival and risk factor analysis

**Fig. 2:**
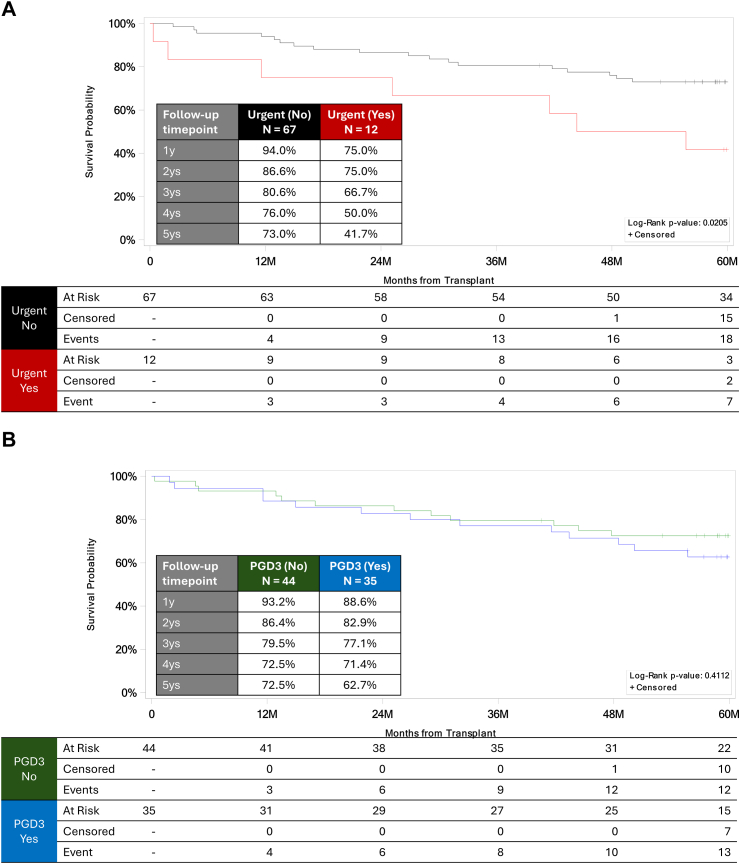
Overall survival in the EXPAND cohort. (A) Kaplan–Meier graph showing survival in urgent (red line) and non-urgent (black line) patients. Urgent = Yes is defined as LAS ≥50 (for US patients) or High Urgency status (for the Belgian center). (B) Kaplan–Meier graph showing survival in patients with (blue line) or without (green line) primary graft dysfunction grade 3 (PGD3) within 72 h.

Urgency of transplant was significantly associated with mortality, as shown by the Kaplan–Meier method (log-rank *P* = 0.0205; Fig. 2A), univariate analysis (9.3% vs 28.0%) (Table 2), and Cox regression analysis (hazard ratio [HR] 3.023, 95% confidence interval [CI] 1.209–7.554, *P* = 0.018) (Table 3). Five-year overall survival was much greater for patients designated as non-urgent than for patients designated as urgent (73% versus 41%). Of note, donor and recipient factors did not differ significantly between the urgent (n = 12) and non-urgent (n = 67) OCS cohorts except for ventilator status before transplant (Supplementary Table S2). As expected, the urgent cohort had a greater proportion of patients on the ventilator before transplant (25% versus 0%, *P* = 0.0028). Older recipient age was also significantly associated with 5-year mortality, as shown by Cox regression analysis (HR 1.34, *P* = 0.012) (Table 3).

**Table 2:** Univariate analysis of the OCS EXPAND cohort (N = 79) assessing risk factors for 5-year overall survival.

	Statistics	Alive at 5 y N = 54	Died within 5 y N = 25	*P*
Donor risk factors				
Donor age, y	mean ± std (min–max)	46.8 ± 15.9 (17.0–76.0)	47.8 ± 17.0 (16.7–69.0)	0.7963
Donor BMI, kg/m^2^	mean ± std (min–max)	26.9 ± 5.7 (16.9–41.8)	27.3 ± 6.4 (18.1–44.1)	0.7750
Donor gender—female	n/N (%)	21/54 (38.9%)	12/25 (48.0%)	0.4716
PaO_2_/FiO_2_	mean ± std (min–max)	371.8 ± 107.5 (135–624)	390.1 ± 116.2 (192–633)	0.4974
Recipient risk factors				
Recipient age, y	mean ± std (min–max)	53.5 ± 11.3 (31.8–73.7)	60.0 ± 7.1 (42.0–68.2)	0.0027
Recipient BMI, kg/m^2^	mean ± std (min–max)	24.6 ± 4.6 (16.4–33.6)	24.4 ± 4.5 (16.2–33.4)	0.8592
Recipient gender—female	n/N (%)	20/54 (37.0%)	13/25 (52.0%)	0.2298
Urgent status	n/N (%)	5/54 (9.3%)	7/25 (28.0%)	0.0441
LAS	mean ± std (min–max)	40.2 ± 10.7 (31–91)	45.5 ± 17.4 (32–93)	0.1806
ECMO	n/N (%)	1/54 (1.9%)	0	1.000
Ventilator support	n/N (%)	1/54 (1.9%)	2/25 (8.0%)	0.2339

BMI body mass index, ECMO extracorporeal membrane oxygenation, LAS lung allocation score, OCS Organ Care System. *P*-values were calculated with the two-sample t-test for continuous variables and the Fisher Exact test for categorical variables.

**Table 3:** Cox regression analysis for 5-year mortality in the EXPAND cohort.

Risk factors	Hazard ratio	95% CI	*P*
Urgent status (Yes vs No)	3.023	[1.209–7.554]	0.0180
Donor age (/5 y)	0.960	[0.833–1.107]	0.5758
DCD donor (Yes or No)	0.889	[0.357–2.210]	0.7997
Donor gender (Female vs Male)	1.268	[0.527–3.051]	0.5956
Donor PaO_2_/FiO_2_ (/unit)	1.002	[0.998–1.006]	0.3218
Recipient age (/5 y)	1.340	[1.065–1.685]	0.0123

DCD donation after cardiac death. *P*-values calculated by Cox regression method.

**Supplementary Figure S1:**
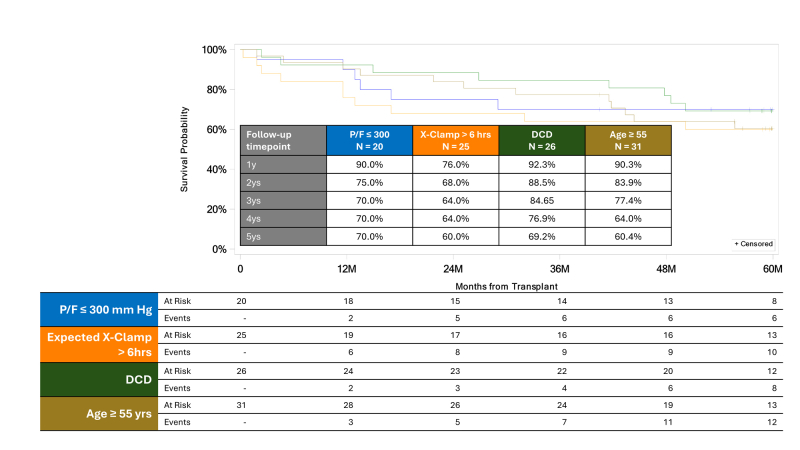
Post hoc Kaplan-Meier lung-recipient survival (EXPAND cohort) stratified by four ECD risk factors.

Five-year mortality was not independently predicted by PGD3 at 72 h either in the unadjusted Kaplan–Meier model (log-rank *P* = 0.4112; Fig. 2B) or in the Cox regression model (HR 1.387, 95% CI 0.633–3.040, *P* = 0.4136). Notably, in the original EXPAND analysis, the incidence of PGD3 within 72 h was higher than in prior reports on SCD lungs, largely because of a higher-than-expected rate of PGD3 at T0 (4–8 h after reperfusion).^9^^,^^10^ No difference was found in overall survival by the individual ECD characteristics (ie, age ≥55 years, PF ≤ 300, anticipated ischemic time >6 h, DCD) (Supplementary Fig. S1).

### EXPAND versus control cohorts

**Fig. 3:**
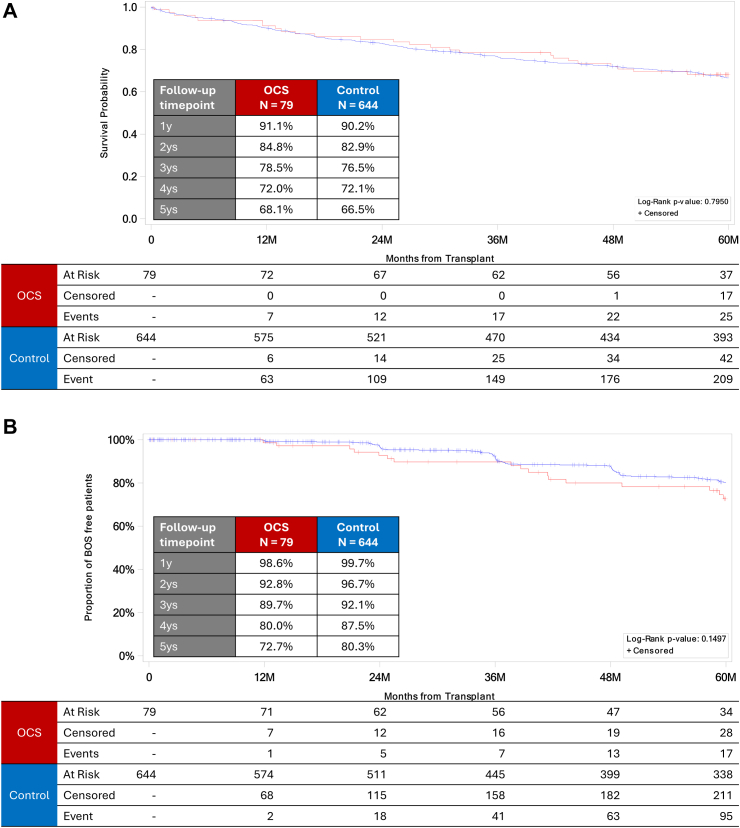

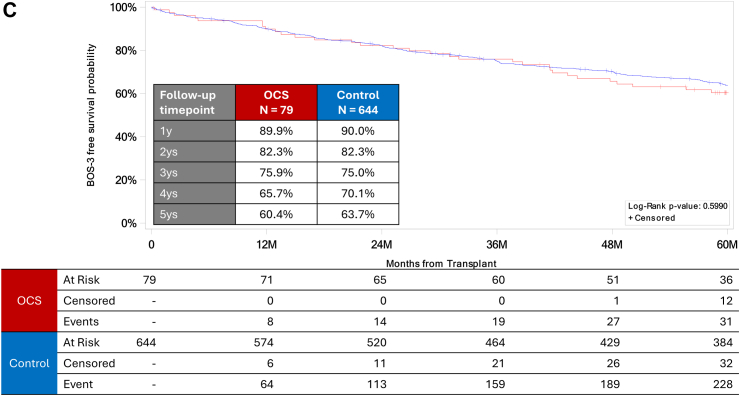
Outcomes in the EXPAND (ie, OCS; red line) versus control (blue line) cohorts (unadjusted benchmark comparison). Kaplan–Meier curves are shown for (A) overall survival, (B) freedom from any grade of bronchiolitis obliterans syndrome (BOS), and (C) freedom from BOS Grade 3 (BOS3) and death.

Overall survival was similar between the EXPAND and control cohorts (Fig. 3A); 5-year overall survival was 68.1% versus 66.5% (*P* = 0.7950), respectively. Likewise, freedom from any BOS (with death censored) was similar between the two cohorts (Fig. 3B), as was BOS3-free survival (Fig. 3C). Five-year BOS3-free survival was 60.4% for the EXPAND cohort and 63.7% for the control cohort (*P* = 0.5990). We also compared freedom from BOS3 between the OCS and control cohorts by using the Fine and Grey method and found that the OCS group had a non-significantly higher 5-year BOS3 rate (HR 1.437, 95% CI 0.603–3.422, *P* = 0.4132).

We assessed risk factors for 5-year mortality in the OCS EXPAND and control cohorts combined, using both univariate and Cox multivariate regression analysis (Tables 4 and 5). On both univariate and multivariate analysis, older donor and recipient ages were associated with 5-year mortality, but urgent status and use of OCS were not. The hazard ratio for use of OCS versus control in the multivariate analysis was 0.805 (95% CI 0.503–1.288, *P* = 0.3653).

**Table 4:** Univariate analysis of 5-year overall survival in the OCS EXPAND and control cohorts combined (N = 723).

	Statistics	Alive at 5 y N = 489	Died within 5 y N = 234	*P*
Donor risk factors				
Donor use of OCS	n/N (%)	54/489 (11.0%)	25/234 (10.7%)	>0.99
Donor age, y	mean ± std (min–max)	36.8 ± 15.0 (10–76)	40.4 ± 16.0 (9–77)	0.0041
Donor BMI, kg/m^2^	mean ± std (min–max)	26.6 ± 6.2 (16.1–57.3)	26.9 ± 6.4 (16.5–66.0)	0.5209
Donor gender—female	n/N (%)	180/489 (36.8%)	96/234 (41.0%)	0.2881
PaO_2_/FiO_2_	mean ± std (min–max)	432.0 ± 116.6 (56.0–1208.0)	423.7 ± 125.6 (77.0–1497.0)	0.3783
Recipient risk factors				
Recipient age, y	mean ± std (min–max)	53.7 ± 13.1 (20–75)	57.1 ± 12.7 (20–76)	0.0008
Recipient BMI, kg/m^2^	mean ± std (min–max)	24.7 ± 4.3 (15.5–36.1)	25.1 ± 4.3 (16.2–35.8)	0.2436
Recipient gender—female	n/N (%)	203/489 (41.5%)	91/234 (38.9%)	0.5183
Urgent status	n/N (%)	148/488 (30.3%)	82/234 (35.0%)	0.2322
LAS	mean ± std (min–max)	48.5 ± 17.9 (28.4–95.0)	50.7 ± 19.3 (32.0–94.4)	0.1371
ECMO	n/N (%)	31/489 (6.3%)	20/234 (8.6%)	0.2806
Ventilator support	n/N (%)	35/489 (7.2%)	24/234 (10.3%)	0.1906

BMI body mass index, ECMO extracorporeal membrane oxygenation, LAS lung allocation score, OCS Organ Care System. *P*-values were calculated with the two-sample t-test for continuous variables and the Fisher Exact test for categorical variables.

**Table 5:** Cox regression model for patient mortality.

Risk factors	Hazard ratio	95% CI	*P*
Actual treatment (OCS vs SOC)	0.805	[0.503–1.288]	0.3653
Urgent status (Yes vs No)	1.151	[0.851–1.559]	0.3615
Donor age (/5 y)	1.057	[1.012–1.104]	0.0133
DCD donor (Yes vs No)	1.318	[0.767–2.266]	0.3178
Donor gender (Female vs Male)	1.087	[0.831–1.421]	0.5439
Donor PaO_2_/FiO_2_ (/unit)	1.000	[0.999–1.001]	0.6359
Recipient age (/5 y)	1.091	[1.030–1.156]	0.0031
Pre-Tx ECMO (Yes vs No)	1.062	[0.565–1.997]	0.8520
Pre-Tx ventilator support (Yes vs No)	1.409	[0.794–2.502]	0.2415

DCD donation after cardiac death, ECMO extracorporeal membrane oxygenation, OCS Organ Care System, Pre-Tx pretreatment. *P*-values calculated by Cox regression method.

### Subgroup analysis of EXPAND versus control cohorts

**Fig. 4:**
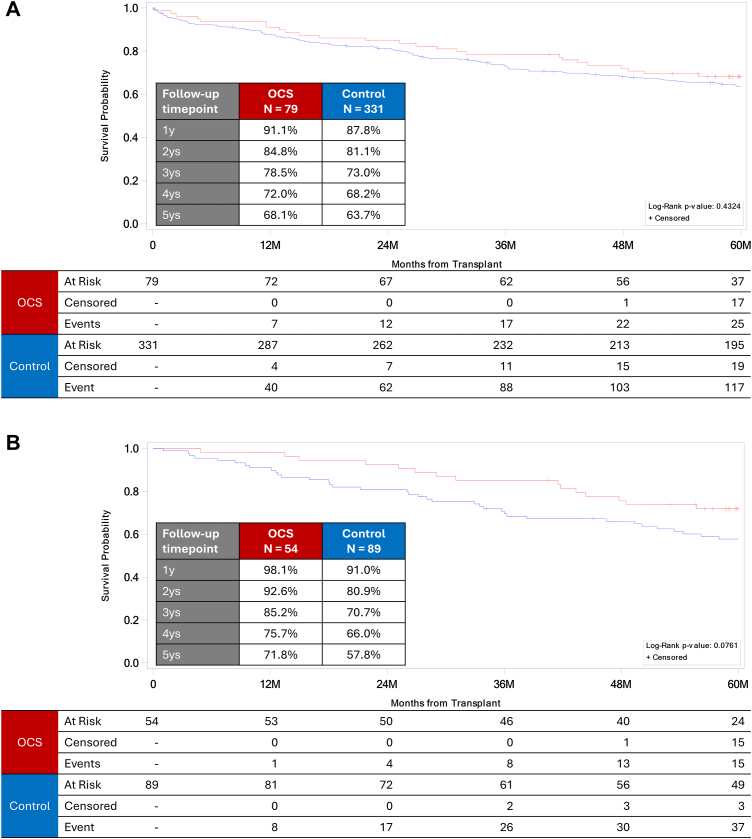
Unadjusted subgroup analysis comparing overall survival in the EXPAND cohort (ie, OCS; red line) versus the ECD subgroup of the control cohort (blue line). All cases had at least 1 ECD factor; in the two analyses, cases with expected ischemic time >6 h in the EXPAND cohort and actual ischemic time >6 h in the control cohort were either (A) included or (B) excluded. Thus, only cases with older recipients, low-PF recipients, and DCD donors were included in the second subgroup analysis *[(Extended criteria donor, ECD = DCD, PF <300, age >55 y, anticipated ischemic time >6 h (in OCS group), actual ischemic time >6 h (in control group)].*

As expected, donor and recipient factors differed between the EXPAND and control cohorts. However, we were able to compare outcomes between subgroups of patients in four separate analyses. First, a subgroup of 331 control patients was identified who had at least one of the four EXPAND ECD criteria, including total ischemic time >6 h. In this cohort, the actual ischemic time was used as a surrogate for expected ischemic time, which was the inclusion criterion used in the EXPAND trial. In this unadjusted analysis, overall survival did not differ between the EXPAND and ECD control subgroups (Fig. 4A).

Next, we excluded EXPAND and control ECD patients who had expected or actual (respectively) ischemic times >6 h. This left 54 patients in the EXPAND subgroup and 89 patients in the control ECD subgroup. An unadjusted comparison between these two subgroups suggested that the controls trended toward poorer overall survival (*P* = 0.0761) (Fig. 4B).

**Supplementary Figure S2:**
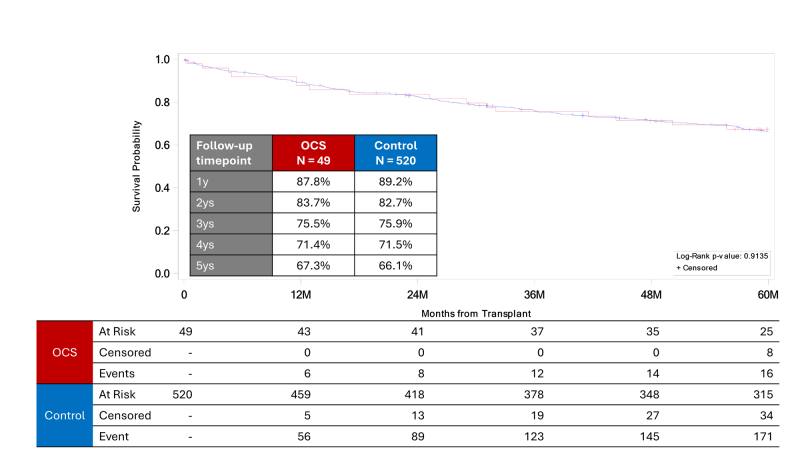
Kaplan-Meier survival in 79 pairs of propensity-score-matched EXPAND and control patients. Propensity matching adjusted for recipient age, urgent status, extracorporeal membrane oxygenation (ECMO), and ventilator support.

Subsequently, we compared unadjusted 5-year overall survival between the EXPAND (n = 49) and control lung recipients (n = 520) with LAS ≥35; it was not possible to use an LAS cutoff greater than 35 without making the EXPAND group too small for a meaningful comparison. No difference in overall survival was noted between these two groups (Supplementary Fig. S2).

**Supplementary Figure S3:**
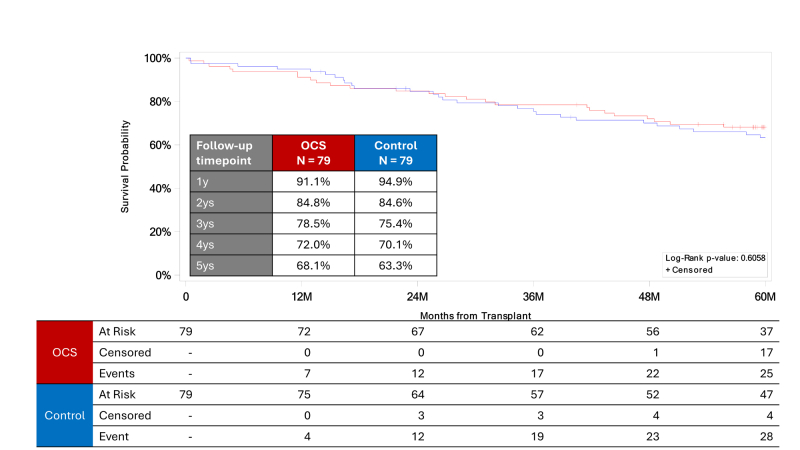
Patient survival in the EXPAND cohort (ie, OCS; red line) versus a control cohort (blue line) comprising a subset of US patients with a lung allocation score (LAS) ≥35.

Finally, in a confirmatory analysis, we used propensity score matching to identify a control cohort (n = 79) with similar recipient age, urgent status, ECMO, and ventilator support as the EXPAND cohort (n = 79). In the control group, 565 patients were excluded after propensity score matching. The characteristics of donors and recipients are summarized in Supplementary Tables S3A and B with standardized mean difference and *P*-values. The risk factors used for propensity score derivation were balanced between the two groups, although the OCS group still had a greater mean donor age and a lower mean donor PaO_2_/FaO_2_ ratio. Five-year overall survival was similar between the two groups (*P* = 0.61) (Supplementary Fig. S3).

## Discussion

EVLP is among the most promising innovations in lung transplantation for expanding the donor pool, but long-term multicenter evaluations of its use in clinical practice are lacking. Encouragingly, Divithotawela and colleagues reported single-center outcomes from the Toronto Lung Transplant Program showing comparable rates of CLAD at 9 years between patients who received lungs preserved with static EVLP or conventional cold storage.^20^ Retrospective studies using the UNOS database have explored early and late outcomes of EVLP in a multicenter setting. Chen and colleagues reported the outcomes of 345 EVLP transplants, showing 90% 6-month survival and 75% 2-year survival.^21^ Jawitz and colleagues reported the outcomes of 155 EVLP transplants, showing 92% 6-month survival.^22^ These analyses provide important real-world data on EVLP usage but lack the focus, standardization, and control over data collection provided by a prospective cohort study design, which is integral to the evaluation of novel technologies by regulatory agencies.

Two prospective multicenter studies have evaluated the effects of static EVLP on mid-to long-term outcomes after lung transplant in the United States. Mallea and colleagues reported 89% survival and 96% freedom from BOS at 1 year in 66 recipients of lungs preserved using static EVLP monitored remotely by perfusion centers.^23^ Gouche and colleagues reported 64% survival and 90% freedom from BOS at 3 years in 110 recipients of lungs preserved with the static XPS EVLP system (XPS, XVIVO Inc).^12^

The EXPAND trial was the first multicenter, international clinical trial to evaluate the use of portable normothermic EVLP with the OCS Lung for the preservation of ECD lungs. The results of this study were published in 2019, and although the study included European centers (Germany and Belgium), the cohort was predominantly American.^10^ The EXPAND follow-up study reported here was a post-approval study required by the US Food and Drug Administration (FDA) to determine overall survival and the 5-year incidence of BOS in the original EXPAND cohort.

This study had three principal findings. First, 5-year overall survival and 5-year freedom from any BOS in the EXPAND cohort were 68.1% and 72.7%, respectively, and were comparable to those of the controls (66.5% and 80.3%). Second, BOS3-free survival at 5-year follow-up was 60.4% in the EXPAND cohort, which was also comparable to that seen in the control cohort (63.7%). Third, the only risk factors associated with 5-year mortality in the EXPAND cohort were the recipient's age and urgency for lung transplantation.

Most lung transplants use SCD lungs rather than lungs with ECD features,^1^^,^^2^^,^^15^^,^^17^ as evidenced by the fact that only 15% of the donors in our control cohort were older than 55 years, only 8.7% had a PF < 300, and only 3.3% were DCD donors. Despite the low number of ECD lungs used for transplant, these organs comprise the majority of donor offers made to transplant centers.^1^^,^^7^^,^^17^ This discrepancy was evident in the EXPAND trial, in which a review of the UNOS-OPTN match run data for the US sites showed that 66 donor lungs perfused on OCS and transplanted were initially turned down by transplant centers a mean of 33 times (range 0–197).^10^ The EXPAND trial had the highest donor utilization rate reported in a multicenter evaluation of EVLP-preserved ECD lungs.^10^^,^^11^^,^^24^^,^^25^ In addition, the EXPAND cohort had more than twice as many donors with multiple ECD criteria as the control cohort (27% versus 12%).

PGD3 within 72 h in the EXPAND trial was more frequent than expected due to the relatively high incidence of PGD3 at time zero (41%). Despite prior evidence showing an association between PGD3 and poor long-term graft and patient outcomes,^26^, ^27^, ^28^ PGD3 was not associated with overall survival in the EXPAND study. One possible explanation relates to the duration of PGD3 in that study: The PGD3 rates at 48–72 h were no different than those for SCD lungs.^9^^,^^10^^,^^29^ PGD3 that persists at 48–72 h has greater discriminant power for predicting long-term mortality than PGD3 at earlier time points.^30^ Thus, it is conceivable that PGD3 in the EXPAND trial was not associated with overall survival because most of the PGD3 was transient and did not last until 48–72 h. It is also possible that the study was underpowered to detect an association between PGD3 and overall survival in this cohort. Alternatively, it is possible that the PGD3 phenotype observed after EVLP has a different pathophysiology than the PGD3 observed after standard ice storage, such that EVLP-related PGD3 is less injurious in the long term than ice-storage–related PGD3. Consistent with this hypothesis, others have found that PGD3 in lungs exposed to EVLP does not correlate with poorer survival, as one would expect for standard ice preservation.^12^^,^^23^ EVLP exposes the donor lung's endothelium to blood flow throughout preservation, which could cause gaps in the vascular lining and result in early fluid shifts that mimic the diagnosis of PGD3 after reperfusion. Yet, the endothelial mechanisms behind this pulmonary edema may differ substantially from the innate immune-based mechanisms pathognomonic of PGD3.^31^^,^^32^ Clearly, additional molecular and clinical investigation is required to clarify the differences between reperfusion after EVLP and reperfusion after ice preservation.

Not surprisingly, in the EXPAND trial, like previous studies, the recipient's transplant urgency was independently associated with overall survival.^1^^,^^2^^,^^22^ Donor lung allocation is determined largely by the degree of urgency for transplant; thus, a patient's transplant center is more likely to receive and be able to accept a standard donor offer if the patient is in urgent need of transplant. This also suggests that a pool of donors is needed for lower-acuity patients to reduce the chance of waitlist mortality. ECD lungs perfused with OCS Lung could fill this void, producing outcomes similar to those associated with SCD lungs. But it is not clear whether ECD lungs perfused with OCS must be used in patients with greater urgency if doing so poses a risk to the patient, and if the patient is already prioritized to receive SCD lungs. Certain factors such as small stature, sensitization, blood type, and geographic location can still result in a patient waiting a long time for a suitable donor lung despite having high urgency for transplant. The updated allocation system—the Composite Allocation Score (CAS) system, implemented in March 2023—aims to address some of these disparities, but the EXPAND trial predates this system. It is possible that the observed effect of urgency on overall survival in the OCS cohort was more an artifact of the sample size than a true effect. This is evidenced by the fact that the multivariate analysis of the combined cohort found no association between the urgency of transplant and 5-year mortality. In addition, 5-year overall survival among patients with LAS ≥35 did not differ between the OCS cohort and the control cohort.

Several single-center and multicenter studies have demonstrated acceptable short- and long-term outcomes with standard ice preservation of lungs from donors with ECD features such as older age, low PF ratios, expected ischemic times >6 h (usually associated with longer geographic distances between donor and recipient), and DCD.^2^, ^3^, ^4^^,^^8^ However, these studies have several limitations, including retrospective study designs, highly selected patient cohorts, and challenges in collecting and reporting early graft dysfunction rates. These limitations may, in part, underlie the reluctance within the transplant community to fully embrace the use of ECD lungs.^1^^,^^5^^,^^7^^,^^33^^,^^34^ Valapour and colleagues’ most recent 2022 OPTN update showed that ECD lungs remain vastly underutilized: Only 7.4% of transplants used DCD donors, and only 11.9% used lungs from donors >55 years old.^35^

Overall survival did not differ between the EXPAND cohort and the ECD subgroup of the control cohort. This supports prior evidence that ice preservation can be safe for ECD lungs. However, this was only a subgroup analysis, and the donors in the control cohort were probably highly selected for specific recipient indications. Nonetheless, it raises the important point that OCS technology, which adds an operating expense of up to $60,000 per case, may not be necessary in all ECD lung transplants. The current study did not intend to resolve the question of OCS's superiority over standard ice for all ECD lungs, but it provides reassurance that ECD lungs preserved with the OCS Lung have good short- and long-term outcomes. We did perform additional subgroup analyses to determine whether there were subpopulations that showed better overall survival with OCS versus standard lung preservation. These analyses were complicated by the fact that OCS was nearly always associated with substantially longer out-of-body times than static cold storage. In addition, as the groups for comparison become smaller, the study loses power to produce definitive findings.

It is currently unclear whether perfusion time on the OCS Lung should be considered in the designation of ECD, because the organ is continuously perfused with blood. Therefore, we compared EXPAND and control subgroups with ischemic times of ≤6 h to remove the potentially confounding element of expected ischemic time. In this subgroup comparison with only DCD, older age, or low-PF cases, overall survival appeared better in the EXPAND group. A randomized controlled trial of EVLP versus ice would be the only way to definitively establish the difference in outcomes between the two preservation methods for ECD organs; however, for ethical and logistical reasons, such a trial may never be conducted. On the other hand, emerging static ice preservation platforms such as the LungGuard (Paragonix) and the XPS static EVLP system (XVIVO) could provide an important comparator group for such an investigation.^23^^,^^36^

Some investigators have expressed concern over the use of EVLP with ECD lungs for fear of exacerbating inflammation and PGD.^11^ Thus, it is worth considering whether OCS could worsen the function of an ECD organ and whether the suggested EVLP parameter thresholds for making decisions regarding transplantability are sufficient for ensuring the quality of the donor lung for transplant.^11^ Early data from the EXPAND study showed that of the 12 lungs that were refused for transplant, six were refused because of evidence of significant contusions or visible air leaks.^10^ This accounted for 6% of the 93 donor lungs screened in the trial. For an extended-criteria cohort, this non-use rate compares favorably with the 9% rate documented in Valapour and colleagues' 2022 OPTN report for all donor lungs in the United States.^35^ Contusions and air leaks can result from procurement trauma or develop as part of the donor organ's evolving pathology, and lungs with these characteristics are typically not recommended for ex vivo perfusion and ventilation. Regarding parameters, both short-term and long-term follow-up studies provide confidence in the suggested thresholds for transplantability, which include PF > 300 and stable physiologic parameters. Future studies will need to resolve whether outcomes are even better if higher PF thresholds for transplant are used, but that benefit may come at the expense of decreased donor utilization. Certainly, precision-based medicine using biomarkers from the perfusate will be an important area of discovery in this field to avoid the subjectivity of gross donor lung evaluation by a surgeon. Lastly, the National Organ Perfusion (NOP) program aims to reduce subjectivity and the margin for error in OCS Lung procurements by deploying specialized teams dedicated to OCS perfusion.^37^

This study has limitations. All controls’ BOS data were obtained from the OPTN registry, to which the reporting of these data may vary among centers. However, BOS3 represents an objective and severe decline in donor lung function and thus provided a consistent indicator of long-term graft dysfunction across sites. As noted in the Study Design section, the incidence of CLAD was not analyzed because data were inconsistent across the study period and the participating institutions, as well as the fact that BOS, not CLAD, was the prespecified long-term graft-related endpoint at the start of the EXPAND trial.^14^ We did not collect data related to individual immunosuppression protocols, which could influence BOS incidence and BOS3-free survival. However, we did collect data on acute rejection within 30 days, the rate of which was 0% in the EXPAND cohort and 2% in the INSPIRE standard ice cohort.^10^ Recipient factors differed between the EXPAND and control cohorts in the benchmark analysis, particularly in terms of urgency of transplant. This discrepancy was probably a result of the observed tendency to allocate lungs to lower-risk recipients in the early portion of the EXPAND trial period.

Nonetheless, a comparison between propensity-matched OCS and control patients and a subgroup comparison of patients with LAS ≥35 both showed similar overall survival between the two groups. Importantly, this was an observational study without a formal power analysis to determine the number of patients needed to detect a difference in outcomes between groups. Thus, a larger sample size could conceivably reveal a difference in favor of either the OCS or the control cohort. However, even if there were a 5% survival difference between OCS and controls at 5 years (60% versus 65%), the study would require around 1000 patients per group (60% power) to produce conclusive results. Furthermore, the control population in this study was limited to US centers through our agreement with the OPTN; we did not have access to control cases in the European centers. Finally, the EXPAND trial was conducted before the allocation system, known as the CAS system, was updated in 2023. This system aims to reduce disparities in donor lung allocation and potentially increase the number of available SCD lungs for high-urgency recipients. However, initial findings suggest that implementing the CAS system has led to longer travel and organ ischemic times.^38^ This may make portable OCS technology, designed for long-distance retrievals, more favorable than cold storage, although this has yet to be fully evaluated. The benefit of OCS Lung use in the CAS era is currently being assessed in the Thoracic Organ Preservation registry.^39^

In summary, the use of the OCS Lung to perfuse and ventilate ECD lungs in the EXPAND trial was associated with excellent long-term overall and BOS3-free survival. The EXPAND trial provides high-level evidence supporting the safety of using portable normothermic EVLP for the transportation, preservation, and monitoring of ECD lungs before transplant. The benchmark comparison, however, raises the possibility that certain ECD lungs, particularly those with shorter total cross-clamp times, can produce similar outcomes to those achieved using OCS technology. The current study indicates that lungs with longer transportation times or that may benefit from ex vivo evaluation before implantation can be safely considered for portable EVLP with the OCS Lung. This approach could be especially valuable in patients with lower urgency or other barriers to receiving a donor organ promptly. The expanded use of ECD lungs has the potential to increase the donor pool for lung transplantation, offering hope to patients with irreversible lung disease who are awaiting a life-saving organ transplant.

## Contributors

All authors participated in the conception, design, and conduct of the study, including data entry oversight, patient recruitment, interval analyses, designation of clinical outcomes, manuscript writing, and final approval of the manuscript. Drs Gabriel Loor and Marshall Hertz accessed and verified the data used for analysis in this study.

## Data sharing statement

Data sharing requests will be considered from research groups that submit a research proposal and an appropriate statistical analysis and dissemination plan to the sponsor, Transmedics, Inc., at datarequrest@transmedics.com. Data will be shared via a secure data access system once the request is reviewed by the steering committee.

## Declaration of interests

Funding for the EXPAND trial and the long-term follow-up study came from Transmedics. Transmedics was not involved in the authorship of the manuscript except that Xiang Zhou, PhD, performed the analyses for this manuscript. The analysis plan was specified by the study investigators.

Gabriel Loor: Paid consultant for Transmedics National Organ Care System Program (NOP) (2022–2024), Steering Committee co-chair for the Transmedics Thoracic Organ Perfusion (TOP) Registry, former consultant for Abiomed (2020), institutional grant support for research from Transmedics, Abiomed, JLH Foundation, Abbott, and Atricure. Founder and CMO of OrganVive, LLC.

Mani A. Daneshmand: Received meeting travel/attendance support from AbioMed. Board member for Procure on Demand.

Matthew Hartwig: Steering Committee member for National Organ Care System Program (NOP).

Marshall Hertz: Steering Committee co-chair for Transmedics Thoracic Organ Perfusion (TOP) Registry. Receives grants or contracts from Transmedics, Cystic Fibrosis Foundation, Biomedinnovations, and Paragonix. Receives consulting fees from CSL Behring, Transmedics, Intuitive Surgical, Paragonix, and Lung Bioengineering.

Stephen Huddleston: Institution received research support from Transmedics. Received a mid-career faculty grant from the American Society of Transplant Surgeons.

Joren Madsen: Thoracic Organ Perfusion (TOP) Registry Data Safety Monitoring Board member.

Michael A. Smith: Vice President, Western Thoracic Surgical Association.

Dirk Van Raemdonck: Local site principal investigator for INSPIRE and EXPAND Clinical Trials.

Gregor Warnecke: Institution received research support from Transmedics. Received honoraria and meeting travel/attendance support from AbioMed.

Xiang Zhou: Employed by Transmedics.

None of the other authors has disclosures to make.
