# Supplementary material for: Long-term outcomes of the international EXPAND trial of Organ Care System (OCS) Lung preservation for lung transplantation
Source: eClinicalMedicine. 2025 Jul 8;85:103334. doi: 10.1016/j.eclinm.2025.103334 (PMC12273511; doi:10.1016/j.eclinm.2025.103334)
Supplement: Supplementary Tables [file mmc1.docx]

***Supplementary Table S1:* Comparison of DCD characteristics in the OCS EXPAND cohort and the control cohort.**

|  | **Statistics** | **OCS**  **N = 26** | **Control**  **N = 21** | ***P*** |
| --- | --- | --- | --- | --- |
| Donor age, y | mean ± std  (min – max) | 40.3 ± 13.9  (16.7 – 69.0) | 34.9 ± 15.6  (14.0 – 62.0) | 0.2127 |
| Donor BMI, kg/m^2^ | mean ± std  (min – max) | 25.2 ± 5.6  (16.9 – 36.1) | 27.3 ± 5.4  (17.5 – 39.5) | 0.2049 |
| Total cross-clamp time,^a^ min | mean ± std  (min – max) | 607.9 ± 146.0  (359 – 899) | 346.4 ± 96.7  (204 – 584) | <0.0001 |
| PaO_2_/FiO_2_ | mean ± std  (min – max) | 407.5 ± 80.7  (250 – 624) | 421.3 ± 92.8  (235 – 584) | 0.5902 |
| Warm ischemic time^b^ | mean ± std  (min – max) | 27.6 ± 4.8  (20 – 35) | 27.8 ± 8.4  (18 – 49) | 0.9212 |

BMI body mass index, DCD donation after cardiac death, OCS Organ Care System.

^a^Total cross-clamp time = time from cold pulmonary artery flush to longest reperfusion time in the recipient. ^b^Warm ischemic time = time from withdrawal of life support to pulmonary artery flush. *P*-values were calculated with the two-sample t-test for continuous variables and the Fisher Exact test for categorical variables.

***Supplementary Table S2:* Comparison of donor and recipient demographics between non-urgent and urgent subgroups of the OCS EXPAND cohort (N=79).**

|  | **Statistics** | **Non-urgent – OCS**  **N = 67** | **Urgent – OCS**  **N = 12** | ***P*** |
| --- | --- | --- | --- | --- |
| Donor | | | | |
| Donor age, y | mean ± std  (min – max) | 47.8 ± 16.1  (16.7 – 76.0) | 43.1 ± 16.9  (20.8 – 67.7) | 0.3567 |
| Donor BMI, kg/m^2^ | mean ± std  (min – max) | 26.8 ± 5.7  (16.9 – 44.1) | 28.8 ± 6.9  (18.3 – 38.6) | 0.2788 |
| Donor gender – female | n/N (%) | 27/67 (40.3%) | 6/12 (50%) | 0.5434 |
| CC time, min | mean ± std  (min – max) | 607.6 ± 131.8  (353 – 1047) | 619.3 ± 104.2  (452 – 821) | 0.7716 |
| Ischemic time, min | mean ± std  (min – max) | 234.1 ± 98.3  (70 – 436) | 240.0 ± 79.1  (57 – 401) | 0.8452 |
| Donor PaO_2_/FiO_2_ | mean ± std  (min – max) | 381.4 ± 109.9  (135 – 663) | 357.1 ± 112.9  (144 – 624) | 0.4841 |
| Donor age >55 y | n/N (%) | 25/67 (37.3%) | 6/12 (50%) | 0.5238 |
| Expected CC > 6 h | n/N (%) | 20/67 (29.9%) | 5/12 (41.7%) | 0.5041 |
| DCD donor | n/N (%) | 23/67 (34.3%) | 3/12 (25%) | 0.7412 |
| PaO_2_/FiO_2_ <300 | n/N (%) | 17/67 (25.4%) | 3/12 (25%) | 1.0000 |
| >1 risk factor | n/N (%) | 17/67 (25.4%) | 4/12 (33.3%) | 0.7234 |
| Recipient | | | | |
| Recipient age, y | mean ± std  (min – max) | 55.6 ± 10.7  (31.8 – 73.7) | 55.3 ± 10.5  (32.9 – 67.8) | 0.9296 |
| Recipient BMI, kg/m^2^ | mean ± std  (min – max) | 24.4 ± 4.8  (16.2 – 33.6) | 25.2 ± 2.8  (19.4 – 28.6) | 0.5426 |
| Recipient gender – female | n/N (%) | 29/67 (43.3%) | 4/12 (33.3%) | 0.7519 |
| LAS | mean ± std  (min – max) | 37.3 ± 4.8  (31 – 48) | 67.5 ± 16.6  (50 – 93) | 0.0001 |
| Diabetes | n/N (%) | 14/67 (20.9%) | 5/12 (41.7%) | 0.1477 |
| ECMO | n/N (%) | 0 | 1 (8.3%) | 0.1519 |
| Ventilator support | n/N (%) | 0 | 3 (25%) | 0.0028 |

BMI body mass index, CC cross-clamp, DCD donation after cardiac death, ECMO extracorporeal membrane oxygenation, LAS lung allocation score, OCS Organ Care System. *P*-values were calculated with the two-sample t-test for continuous variables and the Fisher Exact test for categorical variables. ^a^Urgency for US and German centers was determined by an LAS ≥50. At the Belgian center, urgency was determined by the center’s specific criteria for “high urgency” status, including ventilator and/or ECMO support at the time of listing.

***Supplementary Table S3A:* Comparison of donor and recipient demographics considered for propensity score matching in the OCS EXPAND (N=79) and control (N=644) cohorts.**

| **Parameter** | **Statistics** | **EXPAND (OCS) N=79** | **Controls (SOC) N=644** | **SMD** | ***P*** |
| --- | --- | --- | --- | --- | --- |
| Donor age, y | Mean ± SD | 47.1 ± 16.2 | 36.9 ± 15.0 | 0.659 | <0.0001 |
|  | (min-max) | (16.7-76.0) | (9.0-77.0) |  |  |
| Donor BMI, kg/m^2^ | Mean ± SD | 27.1 ± 5.9 | 26.7 ± 6.3 | 0.061 | 0.6172 |
|  | (min-max) | (16.9-44.1) | (16.1-66.0) |  |  |
| Donor gender – female | n/N (%) | 33/79 (41.8) | 243/644 (37.7) | 0.083 | 0.5398 |
| Donor PaO_2_/FiO_2_ | Mean ± SD | 377.7 ± 110.0 | 435.6 ± 119.3 | 0.505 | <0.0001 |
|  | (min-max) | (135.0-663.0) | (56.0-1497) |  |  |
| Recipient age, y | Mean ± SD | 55.6 ± 10.6 | 54.7 ± 13.3 | 0.073 | 0.5024 |
|  | (min-max) | (31.8-73.7) | (20.0-76.0) |  |  |
| Recipient BMI, kg/m^2^ | Mean ± SD | 24.5 ± 4.6 | 24.8 ± 4.3 | 0.077 | 0.5092 |
|  | (min-max) | (16.2-33.6) | (15.5-36.1) |  |  |
| Recipient gender – female | n/N (%) | 33/79 (41.8) | 261/644 (40.5) | 0.025 | 0.9035 |
| LAS | Mean ± SD | 42.0 ± 13.5 | 50.0 ± 18.7 | 0.493 | <0.0001 |
|  | (min-max) | (31.0-93.0) | (28.4-95.0) |  |  |
| Pre-transplant ECMO | n/N (%) | 1/79 (1.3) | 50/644 (7.8) | 0.317 | 0.0332 |
| Ventilator support | n/N (%) | 3/79 (3.8) | 56/644 (8.7) | 0.203 | 0.1886 |
| Urgent status^a^ | n/N (%) | 12/79 (15.2) | 218/644 (33.9) | 0.444 | 0.0005 |

BMI body mass index; ECMO extracorporeal membrane oxygenation; LAS lung allocation score; OCS Organ Care System; SOC standard of care; SMD standardized mean difference. *P*-values were calculated with the two-sample t-test for continuous variables and the Fisher Exact test for categorical variables. ^a^Urgency for US and German centers was determined by an LAS ≥50. At the Belgian center, urgency was determined by the center’s specific criteria for “high urgency” status, including ventilator and/or ECMO support at the time of listing.

***Supplementary Table S3B:* Comparison of donor and recipient demographics after propensity score matching in the OCS EXPAND (N=79) and control (N=79) cohorts.**

| **Parameter** | **Statistics** | **EXPAND (OCS) N = 79** | **Controls (SOC) N = 79** | **SMD** | ***P*** |
| --- | --- | --- | --- | --- | --- |
| Donor age, y | Mean ± SD | 47.1 ± 16.2 | 36.9 ± 14.4 | 0.671 | <0.0001 |
|  | (min-max) | (16.7-76.0) | (13.0-66.0) |  |  |
| Donor BMI, kg/m^2^ | Mean ± SD | 27.1 ± 5.9 | 26.9 ± 6.1 | 0.019 | 0.9042 |
|  | (min-max) | (16.9-44.1) | (16.5-48.8) |  |  |
| Donor gender – female | n/N (%) | 33/79 (41.8) | 32/79 (40.5) | 0.026 | >0.99 |
| Donor PaO_2_/FiO_2_ | Mean ± SD | 377.7 ± 110.0 | 426.2 ± 82.7 | 0.499 | 0.0022 |
|  | (min-max) | (135.0-663.0) | (163.0-618.0) |  |  |
| Recipient age, y | Mean ± SD | 55.6 ± 10.6 | 55.6 ± 10.6 | 0.001 | 0.9962 |
|  | (min-max) | (31.8-73.7) | (32.0-74.0) |  |  |
| Recipient BMI, kg/m^2^ | Mean ± SD | 24.5 ± 4.6 | 25.0 ± 4.5 | 0.11 | 0.4910 |
|  | (min-max) | (16.2-33.6) | (17.1-35.8) |  |  |
| Recipient gender – female | n/N (%) | 33/79 (41.8) | 39/79 (49.4) | 0.153 | 0.4246 |
| LAS | Mean ± SD | 42.0 ± 13.5 | 43.7 ± 13.1 | 0.131 | 0.4272 |
|  | (min-max) | (31.0-93.0) | (32.5-95.0) |  |  |
| Pre-transplant ECMO | n/N (%) | 1/79 (1.3) | 1/79 (1.3) | 0 | >0.99 |
| Ventilator support | n/N (%) | 3/79 (3.8) | 3/79 (3.8) | 0 | >0.99 |
| Urgent status^a^ | n/N (%) | 12/79 (15.2) | 12/79 (15.2) | 0 | >0.99 |

BMI body mass index; ECMO extracorporeal membrane oxygenation; LAS lung allocation score; OCS Organ Care System; SOC standard of care; SMD standardized mean difference. *P*-values were calculated with the two-sample t-test for continuous variables and the Fisher Exact test for categorical variables. ^a^Urgency for US and German centers was determined by an LAS ≥50. At the Belgian center, urgency was determined by the center’s specific criteria for “high urgency” status, including ventilator and/or ECMO support at the time of listing.
